# Measurement and analysis of maxillary anterior teeth color in the chinese population

**DOI:** 10.4317/jced.60170

**Published:** 2023-04-01

**Authors:** Chenglu Ruan, Lin Wang

**Affiliations:** 1 Dental Department, Sanming Integrated Medicine Hospital, China; 2 Department of Product Design, Sanming University, China

## Abstract

**Background:**

To measure the difference in the crown color of the maxillary anterior teeth in the Chinese population, to study its potential regularity, and to provide a reference for the colorimetry of oral anterior teeth restoration.

**Material and Methods:**

Using VITA Easyshade Advance4.0 spectrophotometer (the colorimetric system is CIE-1976-L*a*b*), adult patients who were treated in Sanming Integrated Medicine Hospital, Fujian Province, China, from January 2022 to June 2022 160 patients (88 males, 72 females, aged 20-60 years) were used as the survey subjects, and the L*a*b* of 1/3 of the crowns of 6 anterior teeth (central incisors, lateral incisors, canines) were measured Value, statistical analysis was performed with SPSS 26.0 software.

**Results:**

The mean L* values of maxillary central incisors, lateral incisors, and canines in the Chinese population were: (73.02±4.41), (69.96±4.70), (65.14±4.21); the mean a* values were: (-0.54±4.21) 0.35), (0.22±0.63), (1.40±0.62), and the mean values of b* were: (14.50±3.23), (18.60±3.94), (23.64±3.30). 1. There was no significant difference in L*a*b* value between left and right symmetrical teeth with the same name (*P*>0.05). 2. There was no statistical difference in the L*a*b* value between genders (*P*>0.05), 3. There was a statistical difference in L*a*b* value between different tooth positions (*P*< 0.05). 4. There were significant differences in L*a*b* values in different age groups (*P*< 0.05).

**Conclusions:**

1. The color of the labial crown of maxillary anterior teeth in the Chinese population is related to different age groups and tooth positions but not gender. 2. In the Chinese population, the color of the maxillary anterior teeth on the labial side gradually decreased from the central incisor to the distal end of the dental arch while the chroma gradually increased. 3. With age increase, the L* and a* values of the upper central incisors, upper lateral incisors, and upper canines gradually decrease, and the b* value gradually increases. The teeth became darker, more yellow, and redder. 4. In the clinical colorimetry of the upper anterior teeth, the contralateral tooth with the same name is preferred. Suppose the tooth with the same name is missing at the same time. In that case, when using the adjacent teeth as a reference, the different brightness and chroma between the central incisors, lateral incisors, and canine teeth should be compared. Change trend to determine. 5. A uniform tooth color should not be selected for anterior restoration, and age should be considered when choosing a color for the patient.

** Key words:**Upper anterior teeth, Crown color, CIE-1976-L*a*b*, spectrophotometric colorimeter.

## Introduction

With the development of society, the restoration of teeth by doctors and patients is not only satisfied with the function but also the shape and position of the teeth. The color is also essential (1). Specialists are paying more and more attention to the optical properties of teeth, and the color of the teeth is the most prominent factor in the optical properties (2). Patients’ concern about the color matching of teeth is also an increasingly common phenomenon(3). It is significant to strive to obtain a beautiful and natural restoration color. There are regional differences in natural tooth color in various regions. Analyzing the tooth color of the anterior teeth of the population in a specific area can better guide the clinical evaluation of the tooth color and make the correct decision. Evaluation in order to be able to produce a restoration that is aesthetically pleasing to the patient (4).

The color and appearance of teeth are complex phenomena with many factors, such as lighting conditions, translucency, opacity, light scattering, etc. (5). It is determined by the combination of the intrinsic color produced by the interaction of light with the tooth structure and the presence of external pigments (6). The scattering and absorption of light by enamel and dentin form the inherent color of teeth (7). Since enamel is relatively translucent, the optical properties of dentin play an essential role in determining the overall tone and chroma of teeth(8). The amount of light reflected and absorbed depends on the thickness and translucency of the tissue, and it is clear that the thickness of enamel and dentin affects tooth color (2).

The measurement of tooth color can be performed by various methods, including visual assessment, spectrophotometry, color chart methods, and computer analysis of digital images (9). These methods have been used successfully with dental colorimetry (5). Visual assessment and colorimetric methods are fast, low-cost, and traditional colorimetric methods. However, they are subject to more significant influences such as external light, the patient’s makeup, experience, and visual fatigue, which are inevitable. Potential errors (10,11), digital image colorimetry is affected by the selection of parameters, post-image processing, and other factors (12). Due to the many disadvantages of subjective tooth color measurement, digital instruments have been introduced and continued to evolve since the late 1970s, enabling objective measurements (1). At the same time, spectrophotometers come with a standard light source and can be protected from environmental conditions. , the influence of the operator (13), and has the function of data storage, which is conducive to collecting, recording, and analyzing data and can communicate with technicians more objectively (14).

In this study, the information collected by the VITA Easyshade Advance4.0 spectrophotometer was more accurate than the visual measurement method(15), their accuracy was improved by 33%, and the color correspondence was also improved by 93.3% (16). In addition, the VITA Easyshade4.0 computer colorimeter has strong repeatability and high accuracy (17,18). The accuracy and repeatability of VITA Easyshade reached 92.6% and 96.4%, respectively(19). Therefore, the VITA Easyshade Advance4.0 wireless computer colorimeter was selected as the experimental colorimetric device in this experiment. The measuring head was placed vertically with 1/3 of the tooth crown for data acquisition. Analyze the changing laws of tooth color so as to provide a reference for clinical colorimetry and post-restoration.

The CIE-1976-L*a*b* chromaticity system in VITA Easyshade Advance4.0 software is used for analysis, and the CIE L*a*b* color notation system of CIE-Commission International e de L’eclairage (International Commission on Illumination) is the most commonly used in dental *in vivo* and *in vitro* studies and color characterization (20,21). In this system L*, a*, b* represent the lightness, chroma green-red coordinates (negative a is green, positive a is red) and chroma blue-yellow coordinates (negative b is blue, positive b is yellow), which is a cylindrical color space description, and tooth color can also be described in a cylindrical color space (22). Many researchers have reported using spectrophotometers, computer colorimeters and other instruments to measure the L*, a*, b* values of teeth to describe the color of teeth (23).

Therefore, in this experiment, VITA Easyshade Advance4.0 spectrophotometer was selected for the experiment. The experiment was completed by the author alone. The instrument was calibrated every time a tooth was changed to reduce errors and provide a certain reference for clinical practice. It can provide a certain reference for the colorimetry of upper anterior teeth in China in the future. The equipment is relatively simple, has strong repeatability, high accuracy, and mature technology. It can provide data support for young doctors, clinical teaching, and student clinics.

## Material and Methods

-Experimental subjects

The Medical Ethics Committee approved this study of Sanming Integrated Medicine Hospital, and the experimental procedures and steps met the requirements. In the selection method of the survey objects, 160 adult patients (88 males, 72 females, aged 20-60 years old) were randomly treated in Sanming Integrated Medicine Hospital from January 2022 to June 2022 selected as the survey objects. A total of 969 6 upper anterior teeth were included in the measurement. All patients lived in Sanming, Fujian for more than 5 years. All patients gave informed consent and met the following inclusion criteria: maxillary permanent anterior teeth, normal development, no history of treatment bleaching, no caries lesions, no Restoration and dental treatment were performed, and the exclusion criteria were: abnormal dental development, teeth with fluorosis, teeth with tetracycline, pigmented teeth, calculus, etc., which could not be combined with colorimetric operations (13,24).

The G-Power software was used to determine the sample size required for this experiment. Through literature review, the test level was set to α = 0.05, and the test power was 1-β = 0.85 (25). The results show that the minimum sample size of this experiment is 60 people.

-Colorimetric system and colorimeter

**Figure 1:**
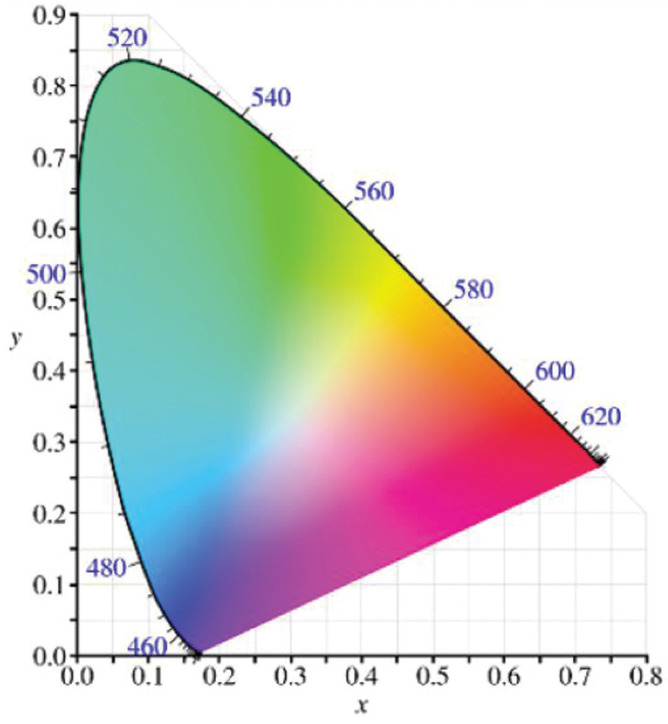
CIE 1976 color space chromaticity diagram (28).

The International Commission on Illumination (Commission Internationale de l’Eclairage, CIE) 1976L*a*b* color space was used for colorimetry (26). L* is the lightness, which means the color is from dark (black) to light (white); a* is the chroma, which means red and green −a*~ a* means the hue changes from green to red, and b* is also the chroma, represents yellow-blue, and −b*~b represents the transition of hue from blue to yellow. The larger the value, the greater the bias (27), (Fig. 1).

Colorimeter: VITA Easyshade Advance4.0 spectrophotometric colorimeter, the instrument comes with a standard D65 light source, and the colorimeter probe diameter is 3mm.

-Experimental Methods

A small brush was used to clean the subject’s teeth, and the upper front teeth were fully exposed. Calibrate the colorimeter. The probes are perpendicular to the incisal, middle, and cervical 1/3 of the tooth surface, respectively, and the top is closely attached to the tooth surface 1/3 to avoid light leakage. After each part is tested three times, the average value is taken, and the next tooth is carried out. Recalibrate the colorimeter when measuring. When comparing data of different age groups with different genders, the 1/3 numbers in the teeth were taken. Data collection was performed by one experimenter to check for operational errors.

-Calculation method of color difference value (14)and inclusion criteria

That is, ΔL=L1-L2; Δa*=a*1-a*2; Δb*=b*1-b*2; total color difference ΔE=(ΔL2+Δa*2+Δb*2)1/2.

The clinically different threshold of total chromatic aberration is ΔE=1.0-3.7, and the clinically accepTable threshold is ΔE=2.72-6.8 (29) If the color difference is smaller than the threshold, it can be considered that the color difference is not recognized by the human eye.

-Statistics Analysis

Spss22.0 statistical software was used to statistically analyze the L*a*b* values of the upper anterior teeth of the population in the Sanming area, and the paired t-test was used for the same name teeth in the same group; the variance of the chromaticity values of different tooth positions and different age groups was analyzed. Analysis, the test level is α = 0.05.

## Results

-Age distribution and number of teeth of the experimental population

After statistics, it was found that the distribution was relatively uniform, including 88 males and 72 females, as shown in Table 1.

**Table 1:**
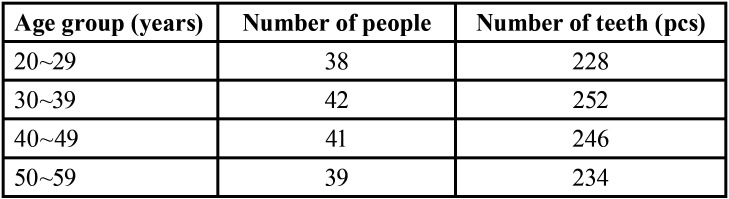
Age and number of teeth of experimental population.

-Chromaticity value of each tooth position

The obtained chromaticity values were statistically analyzed, and it was obtained that each chromaticity value obeyed the ordinary distribution law and was described by the mean ± standard deviation (x±s). The results are shown in Table 2 and Table 3. There was no statistical difference in the chromaticity values of teeth with the same name between different genders (*P* > 0.05).

**Table 2:**
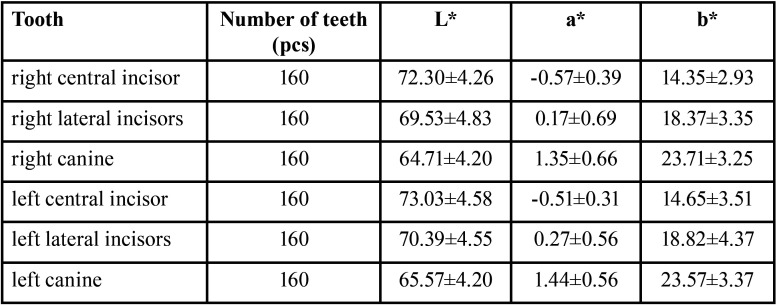
Measurement results of chromaticity value of each tooth position(x±s).

**Table 3:**
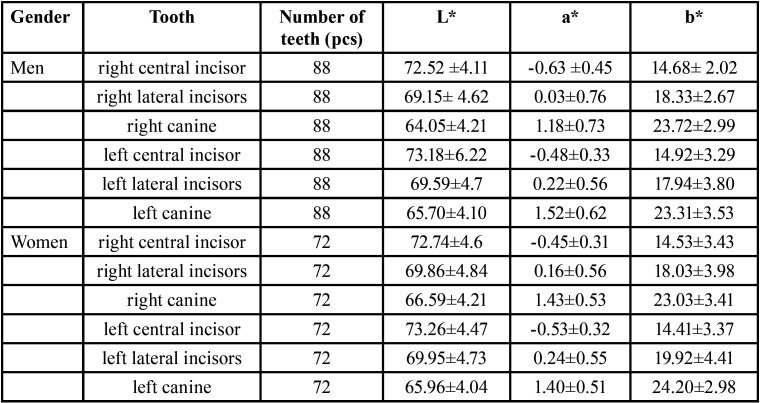
The measurement results of the chromaticity value of each tooth position in different genders(x±s).

-Left-right symmetrical interdental chromaticity values

The L*, a*, b* chromaticity values of the left and right symmetrical teeth with the same name were analyzed by paired t-test, and the results are shown in Table 4. There was no significant difference in the L*, a*, b* chromaticity values of the left and right symmetrical teeth with the same name (*P* > 0.05). Obtain the chromaticity difference (ΔL*, Δa*, Δb*) of the corresponding chromaticity values of the tooth with the same name, and obtain the chromaticity value of the left-right symmetrical tooth with the same name ΔE < 0.9, it can be considered that the color difference is not recognized by the human eye and cannot be recognized by the human eye. The difference in chromaticity between the left and right teeth with the same name.

**Table 4:**
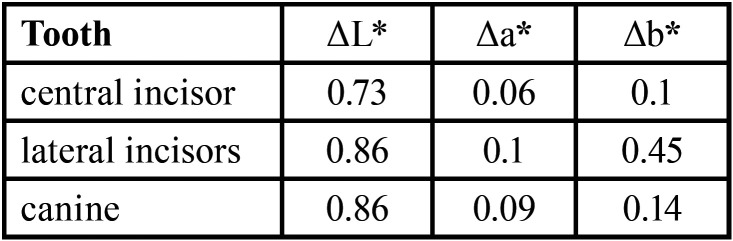
Statistics of chromaticity difference of chromaticity values of left-right symmetrical teeth with the same name.

-Chromaticity values of different tooth positions

The mean ± standard deviation(x±s) description, variance analysis, and paired t-test analysis of the chromaticity values of different tooth positions are shown in Table 5. There was no significant difference in the chromaticity values of the left and right symmetrical teeth with the same name (*P* > 0.05). Among them, 1) The order of lightness L* value is central incisor>lateral incisor>canine; the order of chroma a* value is: canine>lateral incisor>central incisor; the size of chroma b* value The order is: canine>lateral incisor>central incisor.

**Table 5:**
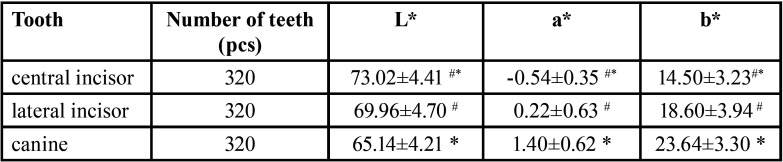
Comparison of chromaticity values of each tooth position (x±s).

Compared with the same group of middle canines, #P < 0.05; compared with the same group of lateral incisors, *P < 0.05

-Comparison of chromaticity values of the same tooth position in different age groups

The mean ± standard deviation (x±s)description, variance analysis, and paired t-test analysis results of chromaticity values L*, a*, b* at different ages are shown in Table 6. L* value between different age groups: L* value of central incisors were significantly different (*P*<0.05); L* value of lateral incisors: there was no statistical difference between the 20-year-old group and the 30-year-old group, and between the 30-year-old group and the 40-year-old group, and there were statistical differences in the other age groups (*P*<0.05); L* value of canine group: there is a statistical difference between the 20-year-old group and the 30-year-old group (*P*<0.05), and there is no statistical difference between the other age groups. A* value between different age groups: The a* value of central incisors: 20-year-old group and 40-year-old group, 20-year-old group and 50-year-old group were statistically different (*P*<0.05), and there was no statistical difference between the other age groups; The a* value of lateral incisors: there was no statistical difference between each age group; a* value of canine teeth: there was a statistical difference between the 20-year-old group and the 50-year-old group (*P*<0.05), and the other age groups were compared in pairs no statistical difference. B* value between different age groups: The b* value of central incisors: the 20-year-old group was significantly different from other groups (*P*<0.05), and there was no statistical difference between the other age groups; The b* value of lateral incisors: there are statistical differences between the 20-year-old group and the 40-year-old group, the 20-year-old group and the 50-year-old group (*P*<0.05), and there is no statistical difference between the other age groups; And canine b* value: there were statistical differences between the 20-year-old group and the 50-year-old group, the 30-year-old group and the 50-year-old group (*P*<0.05), and there was no statistical difference between the other age groups.

**Table 6:**
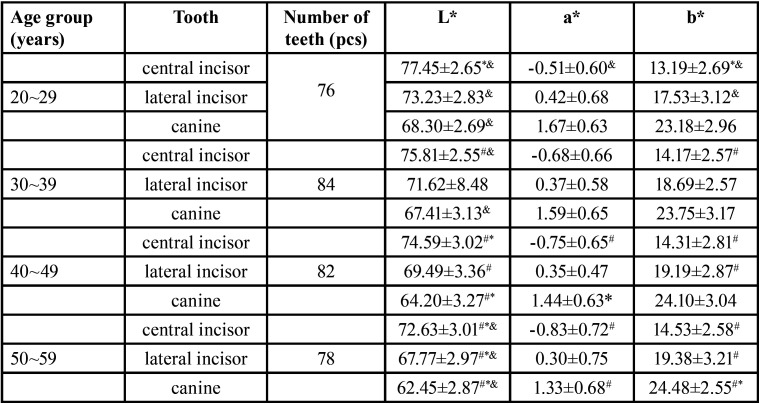
Comparison of chromaticity values for each age group.

Compared with the same tooth position 20-29 years old, #P < 0.05; compared with the same tooth position 30-39 years old, *P < 0.05; compared with the same tooth position 40-49 years old, &P < 0.05

-Mean distribution of different tooth positions in different age groups

**Figure 2:**
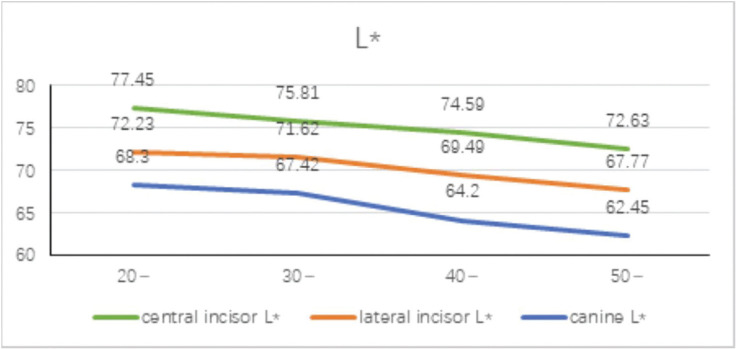
Mean distribution of L* value of different ages and different tooth positions.

The mean distribution of L* values in different tooth positions in different age groups is shown in Figure 2. It can be seen that the L* value tends to decrease with the increase of age. The L* value represents the lightness, indicating that the younger the age, the higher the lightness. As age increases, the value of lightness decreases, and the color develops from white to black. No matter at which age, the L* value decreases from the central incisor to the distal end of the dental arch to the canine, indicating that from the central incisor to the distal end of the dental arch, The lightness gradually decreases from end to canine.

**Figure 3:**
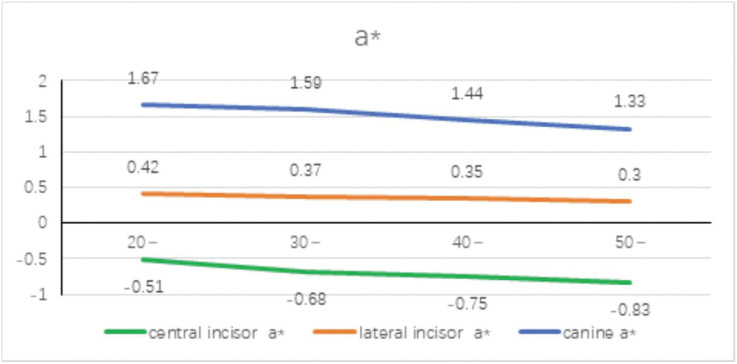
Mean distribution of a* value of different teeth at different ages.

The mean distribution of a* values for different tooth positions in different age groups is shown in Figure 3. It can be seen that the a* value tends to decrease with age. The a* value represents the chroma, representing red and green, and −a*~ a* represents the hue from green to red. With the increase of age, the color develops from red to green. At the same time, no matter at which age, the a* value increases from the central incisor to the distal end of the dental arch to the canine, indicating that from the central incisor to the distal end of the dental arch to the canine Canine chroma develops towards red. With the increase of age, the color develops from red to green. At the same time, no matter at which age, the a* value increases from the central incisor to the distal end of the dental arch to the canine, indicating that from the central incisor to the distal end of the dental arch to the canine Canine chroma develops towards red.

**Figure 4:**
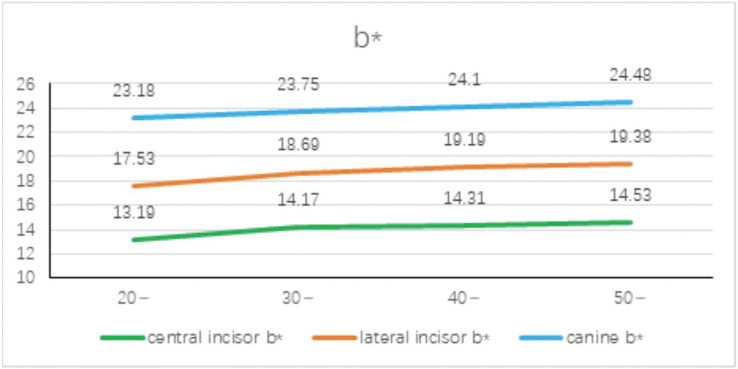
Mean distribution of b* value of different ages and different tooth positions.

The mean distribution of b* values for different tooth positions in different age groups is shown in Figure 4. It can be seen that the b* value tends to increase with age. The b* value represents the chroma, representing yellow and blue, and −b*~b represents the hue from blue to yellow, and the b value increases with age. , the color develops from blue to yellow, no matter at which age the b* value increases from the central incisor to the distal end of the dental arch to the canine, and the color develops from blue to yellow.

## Discussion

This experiment mainly guides the colorimetric work of clinical anterior tooth restoration by studying the natural tooth color and provides a reference for the selection of restoration color. The experimental results show that: 1. The color of the labial crown of the maxillary anterior teeth in the Chinese population is related to different age groups and tooth positions and has nothing to do with gender. 2. The color difference between the left and right teeth with the same name in the Chinese population cannot be recognized by the naked eye, and there is no statistical difference (*P* > 0.05), and the color is very close. 3. In the Chinese population, the color of the maxillary anterior teeth on the labial side gradually decreased from the central incisor to the distal end of the dental arch while the chroma gradually increased. 4. This experiment shows that in the clinical colorimetric analysis of the upper anterior teeth, the control tooth should first choose the same name on the opposite side. If the same tooth on the opposite side is missing at the same time, the adjacent natural tooth can be considered as a reference. At this time, the upper central incisor should be selected. Different brightness and chroma changes between lateral incisors and canines were taken into consideration. 5. The L*a*b* value has nothing to do with gender, and there is no statistical difference (*P* > 0.05). This is consistent with the studies of scholars such as Alma Dozić (30), Yong-Keun Lee (31), Farhad Tabatabaian(32) Tahir Karaman (33). 6. With age, the L* a* values of upper central incisors, upper lateral incisors, and upper canines gradually decrease, and the b* value gradually increases, and the teeth become darker, more yellow, and redder with age. Consistent with Yang DL’s study (34), L* is the coordinate most related to tooth color during aging, which is consistent with the findings of most scholars (35,36). 7. A uniform tooth color should not be selected for anterior restorations, and age should be taken into account when choosing a color for patients, consistent with the Karaman T study (33).

The measurement site selected in this experiment is the middle 1/3 of the crown. Whether there is a difference in the color between the incisal 1/3 of the crown and the neck 1/3 remains to be studied, and this experiment only studies the ipsilateral and The color difference between the contralateral teeth and the difference in brightness and chromaticity between the opposite teeth can be further expanded in future research.
